# The Quality of Life of Former Portuguese Football Players

**DOI:** 10.3390/sports12080200

**Published:** 2024-07-23

**Authors:** Eduardo Teixeira, Carlos Silva, Félix Romero, João Paulo Costa, António Vicente

**Affiliations:** 1 Sport Sciences School of Rio Maior, Polytechnic Institute of Santarém, 2040-413 Rio Maior, Portugal; csilva@esdrm.ipsantarem.pt (C.S.); fromero@esdrm.ipsantarem.pt (F.R.); jpcosta@esdrm.ipsantarem.pt (J.P.C.); 2 Life Quality Research Centre, Polytechnic Institute of Santarem, 2040-413 Rio Maior, Portugal; 3 SPRINT, Sport Physical Activity and Health Research & Innovation Center, 2040-413 Rio Maior, Portugal; 4 Faculty of Human and Social Sciences, University of Beira Interior, 6201-001 Covilhã, Portugal; amnv@ubi.pt; 5 CIDESD, Research Center in Sports Sciences, Health Sciences and Human Development, 5001-801 Vila Real, Portugal

**Keywords:** football (soccer), post-career, former players, quality of life, health

## Abstract

Background: The demands of playing professional football can have an impact on an individual’s quality of life (QoL), which may remain into retirement. Given limited evidence exists regarding the QoL in former football players, this study aimed to assess QoL among Portuguese former players according to career duration, career end period, competitive level, tactical-positional status, international status, academic qualifications, serious injuries in career, and current professional football connection. Methods: The study included 84 Portuguese former football players (48.8 ± 8.2 years old) who transitioned to retirement between 1988 and 2018. The WHOQOL-BREF questionnaire was used to assess QoL perceptions, and the Portuguese version was validated. Results and Discussion: The former players have positive QoL indicators, both in general and across the four domains, namely in terms of the physical, psychological, and social relationship and environment. There were no statistically significant differences in QoL between the defined categories for career end period, competitive level, tactical-positional status, international status, and current professional football connection. Likewise, there was no significant correlation between QoL and career duration. In contrast, there were significant differences in general QoL (*p* < 0.023) and in the physical domain (*p* < 0.001) between former players with different academic qualifications. A significant correlation was found between the number of severe injuries sustained in a career and QoL in the physical domain (R = −0.300, *p* = 0.006). Conclusions: There are no concerning QoL results presented by former players. However, the number of severe injuries sustained during the career was associated with a lower QoL, while holding higher academic qualifications demonstrated higher general and physical QoL. Studies with larger samples should be conducted to confirm these trends.

## 1. Introduction

The concept of Quality of Life (QoL) is closely related to the definition of health. It encompasses an individual’s perception of satisfaction with their daily life, including the impact of physical and psychological health, independence, relationships, personal beliefs and relationships intrinsically linked to the subjective evaluation of individual QoL (1–3)

The World Health Organization (WHOQOL Group) defines QoL as “an individual’s perception of their position in life, within the context of the cultural and value systems in which they are embedded, and in relation to their objectives, expectations, norms, and concerns”. Individuals and their relationships are evaluated subjectively based on characteristics inherent in the respective medium [1,2,3]. The definition of QoL varies depending on the social setting [4] and can be categorized by historical, cultural, and social class stratification [5].

The research on this topic is important in today’s society because it may lead to policy recommendations and actions that promote citizens’ quality of life [6]. Research helps understand and determine if a population’s health conditions affect their quality of life [7]. Despite its ambiguity, QoL may be defined as the value that each citizen assigns to their own position in a society where individuals interact to fulfill their ambitions, needs, and expectations. The general perception of QoL considers the interaction of individual characteristics (needs, values, growth, and expectations) and organizational characteristics (structure, technology, rewards) and their impact on individuals’ psychological and socio-professional development [6,8,9]. These factors include physical and mental health, financial independence, social relationships (family, friends, coworkers, etc.), personal beliefs, and relationships established with the surrounding environment, resulting in general individual satisfaction [10,11]. QoL is an important concept in the field of health and medicine. The majority of QOL studies in health and medicine have conceptual and methodological challenges [12]. The core functions of QoL assessments are identified and related to underlying qualities reflected in the majority of QoL assessments. The attractiveness of these qualities is linked to current trends in philosophical thought, underpinning contemporary culture and social policy [6,13].

Thus, considering the multifaceted impacts of sports participation on QoL, there is a need to consolidate the evidence on QoL in former sports participants. Considering the athletic paradigm, there has been a generalized interest in understanding the impacts of athletic careers [11,14,15,16,17,18,19]. Professional football requires athletes to focus on their athletic performance, sometimes putting QoL as a secondary consideration [14,15,20]. Excessive use of mechanical and energy resources [21,22,23,24,25] high incidence of injuries [26,27] psychological and societal pressures [24,28,29,30] and involuntary career abandonment [11,15,19,31] are some prominent factors that can impact the QoL among football players during short, medium, and long periods of time. 

Compared to non-athletes, transitioning to the post-career stage occurs earlier in life [19,32,33]. This transition marks the end of a professional career and the start of a new phase of life [17,34] resulting in a complex and multidimensional experience [35]. This sensitive period requires players to adjust emotionally to a new social dynamic, status, and lifestyle [36]. Athletes may adopt a sedentary lifestyle after abandoning sports, increasing their risk of developing health issues similar to the general population [15,37]. From another perspective, career experience can provide meaningful memories that improve QoL upon retirement. Factors that promote QoL improvement include peak performance, maximum experience, fluidity, and sublimation, as well as favorable psychological states that provide memories and meaning to life [38]. It is important to understand how former football players see their QoL in the post-career, particularly their satisfaction with their own lives and acceptance of their physical, mental, social, and emotional health [20,39]. The World Health Organization’s Quality of Life (WHOQOL) group [40,41] has promoted research, debate, and discussion on assessing population health and QoL. There are few studies on this topic, especially in the Portuguese context [28]. This situational analysis can provide new indicators and the true impact of professional sports careers on the QoL of football players in the post-career period. Simultaneously, the results can be compared with studies carried out with samples from other countries or even from athletes of other sports. This can contribute to a more in-depth discussion of strategies to reduce the possible negative impact on the professional career.

Football players’ career transitions have been linked to factors such as prioritizing quality and adopting certain lifestyles. Transferring skills such as goal setting, planning, time management, stress management, and energy management are crucial for a successful transition [17,18]. Based on this paradigm, the current study aims to assess the QoL of former Portuguese football players in the post-career stage. The purpose of this study is to compare the QoL of former football players based on factors such as career duration, career end period, competition level, tactical-positional status, international status, academic qualifications, severe career injuries, and current professional connection to football. 

## 2. Methods

### 2.1. Sample 

The sample consisted of 84 former Portuguese football players (age 48.8 ± 8.2 years). A convenience sample was used, taking into account the different regions of residence of former players (Table 1).

Inclusion criteria were former players with at least 8 years of professional experience and a transition to the post-career between 1988 and 2018. Any players retiring after 2018 were not included since they did not have sufficient post-career experience to understand their QoL development, as suggested in the literature [22]. Therefore, to be included in the study, former players had to have at least 3 complete years of retirement.

The participants were informed of the anonymity and confidentiality of the data obtained, and the study was approved by the University of Beira Interior’s Ethics Committee (CE-UBI-Pj-2021-015).

### 2.2. Definition of Variables and Instrument

The dependent variable is QoL, which evaluates the general aspect of QoL and four domains: physical, psychological, social, and environmental. The subjective assessment of QoL was conducted using the World Health Organization Quality of Life questionnaire (WHOQOL-BREF), validated for the Portuguese language [42,43]. The WHOQOL-100, developed by the World Health Organization [40,41], was created to assess QoL from a holistic and transcultural perspective [44]. The WHOQOL-BREF is a valid alternative to the long version, with good psychometric indicators of internal consistency, discriminant validity, construct validity, and test–retest stability, making it a good instrument for assessing QoL in Portugal [42]. It is a useful assessment tool that provides a comprehensive and multidimensional view of the lives of the Portuguese population [7]. The instrument consists of 26 items rated on a Likert scale with five response options. It includes a general QoL and four domains: physical (the individual’s perception of their physical condition), psychological (individual’s perception of their affective and cognitive condition), social relationships (individual’s perception of social relationships and social roles adopted in life), and environmental factors (individual’s perception of different aspects related to the environment in which they live are structured) (Table 2).

The WHOQOL-BREF is a multidimensional and multicultural measure used for the subjective assessment of QoL. The results reflect individual perceptions in each domain [43,45]. Data from the interview guide for the study of the impact of sports careers on the QoL of professional football players in Portugal were used to collect participant information, specifically biographical information from area 1 of the guide [46]. A detailed curriculum vitae was created for each player, including information from the club (and country), division, tactical position, and any internationalization for the national Portuguese team for each season. These were the independent variables included in the study:

- Career duration: Refers to the time span from the first year of professionalization in a senior setting and the final year of professional retirement.
- Career end period: During the 30-year period (1988–2018), two groups of former players were formed: those who retired be-tween 1988 and 2005, and those who retired between 2006 and 2018. The career end period took into account the average number of years of professional abandonment among the 84 individuals surveyed.
- Competitive level: Determined by the divisions in which ex-professional athletes worked during their careers. There are two competitive levels: the 1st division, which includes former players who played more than 50% of career years in the country’s main league, and the 2nd division, which includes players who played more than 50% of career years in the country’s secondary leagues.
- Tactical-positional status: Defined by the tactical position that former players identify as being the one they primarily played during their career. They are divided into four groups according to their position: goalkeepers, defenders, midfielders, and forwards.
- International status: Characterized by the number of internationalizations by the Portuguese “AA” team (Portuguese Football Federation). There are two groups: non-international (zero internationalizations) and international (one or more internationalizations).
- Academic qualifications: Characterized by school-based training for Former players throughout their post-career situation. As a result, three groups were formed with different levels of education: former players with education up to the third cycle (or below), high school education, and university education.
- Severe career injuries: Defined as those caused by trauma or overuse that cause functional impairment in former players [47]. These injuries are considered severe after a month of interruption in training and competition [48]. Injuries were classified based on anatomical location, such as the foot/toe, leg, knee, ankle, thigh, hip/groin, back, head/face/nose and others, as defined by the literature [47,49].
- Current professional connection to football: characterized by direct participation in football that affects monthly earnings. There are two groups: those who have a professional connection to football (e.g., trainers, sports directors, entrepreneurs) and those who do not.

### 2.3. Procedures

Prior authorization was requested from the authors of the psychological instrument via an online request. After obtaining approval, we received the original WHOQOL-BREF version, as well as its user manual and syntax [42,43]. After conducting semi-structured interviews with all study participants and presenting informed consent with explanations of the study’s objectives and purpose, including instructions and response scale descriptions, former players were asked to participate. Data analysis was performed using the Statistical Package for the Social Sciences (SPSS), version BM SPSS Statistics 29. In terms of organization, a syntax was developed to execute supplied data, clean, and compute total points. Next, check the selected options from 26 questions to ensure the correct response interval (between 1 and 5). Later, questions Q3, Q4, and Q26 were recoded (1 = 5; 2 = 4; 3 = 3; 4 = 2; 5 = 1) to better reflect QoL (the questions were negative). The points for each domain were calculated, and the average of the results was multiplied by 100 to compare with the WHOQOL-100 (Table 3).

To characterize the sample and display the points obtained in various domains of QoL perception, descriptive statistics were used (relative frequencies, means, standard deviations, minimum and maximum values). After ensuring data normality, the Pearson Correlation Coefficient was used to measure the correlation between QoL with career duration and the number of severe career injuries. The student *t*-test was used to compare groups for career end period, competitive level, international status and current professional connection to football. A one-way ANOVA was used to compare between groups for tactical-position status, and Tukey’s B post-hoc test was used to locate any significant differences that had been identified. Given the lack of normality in data among groups for academic qualifications, the Jonckheere–Terpstra test was used. This test is a non-parametric alternative to Anova that compares more than two samples when the independent variable is in an ordinal scale [50]. Multiple comparison Pairwise Test was used to verify at what levels differences occur between a continuous dependent variable on a ratio scale and an independent variable on an ordinal scale. All tests were conducted with a significance level of *p* ≤ 0.05.

## 3. Results and Discussion

The study evaluated the QoL of former Portuguese football players, taking into account the general QoL (TWDG) and four domains of analysis: physical (TWD1), psychological (TWD2), social relationships (TWD3), and environment (TWD4). The analysis and discussion of the results focus on comparing the QoL of former players based on their career duration (i), career end period (ii), competitive level (iii), tactical-positional status (iv), international status (v), academic qualifications (vi), severe career injuries (vii), and current professional connection to football (viii).

Former players in the study exhibited positive perceptions of QoL, both in general (78.7 ± 15.7) and in specific domains (Table 4). The average values for each domain range from 79.3 ± 12.3 to 82.09 ± 12.8 points, with the psychological domain being the highest.

The majority of former players perceive a good QoL, contrary to studies that identify professional footballer characteristics as potential causes of QoL decrease in the post-career, specifically, physical demands, injury incidence, psychological and social pressures, among others [19,21,22,23,24,26]. A systematic review of the health conditions of retired football professional players [26] found that this population experiences mental health symptoms such as anxiety and depression, which was not confirmed in the first observation based on psychological results (81.5 ± 12.8). Several studies suggest that physical activity can provide long-term health and QoL benefits [25,51,52]. In a study aimed at the Portuguese adult population, elevated levels of QoL were found, with a focus on physical dominance [7], which may explain some of the findings in this study aimed at football players. To provide a more in-depth analysis, the results obtained from the QoL evaluation of each independent variable included in the study are organized into sub-sections.

### 3.1. Quality of Life and Career Duration

Former players included in the survey had an average professional career duration of 15.2 ± 3.1 years, with a minimum of 8 years and a maximum of 22 years. Pearson’s correlation coefficient shows no statistically significant relationship between QoL and career duration (Table 5). This lack of correlation with career duration is observed in both the general QoL and its various domains (*p* > 0.05).

The duration of former players’ careers is not considered to be related to QoL. The physical domain is highlighted in literature reviews as being influenced by training volume and load, competition, and injuries experienced by athletes during their careers [27,53,54,55]. This fact is closely related to the level of independence that can be lost in the post-career to the level of basic tasks associated with daily activities, such as having autonomy and quality of movement to perform professional or leisure tasks [56]. However, the results obtained did not indicate that individuals with longer careers are likely to have a lower perception of QoL.

### 3.2. Quality of Life and Career End Period

A total of 39.2% of former players finished their careers between 1988 and 2005, and 60.8% between 2006 and 2018. Analyzing QoL scores for former players based on career duration (Table 6) shows that newer individuals have a slightly lower average (78.4 ± 17.3) compared to those who completed their careers earlier (79.1 ± 13.1).

Those who have just completed their careers tend to have a lower general QoL [57], which contradicts expectations regarding long-term health risks and symptoms. It is expected that younger individuals will have higher general perceptions of QoL, as QoL tends to decrease with age. The test does not show statistically significant differences between the two periods, either in the general aspect of QoL or in the remaining domains (Table 7).

**Figure 1:**
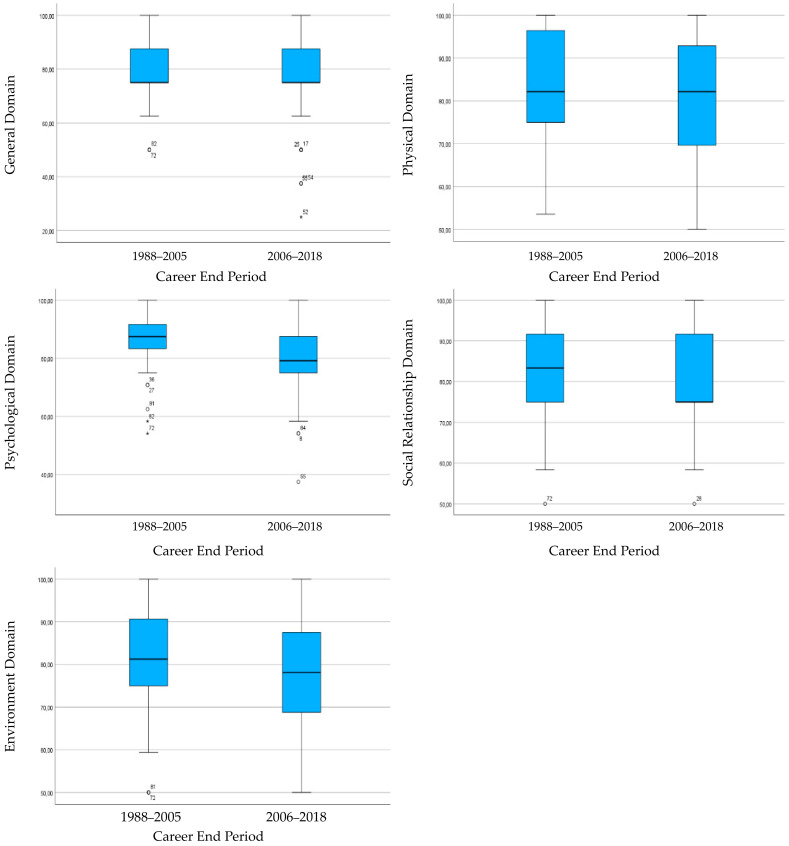
Comparison of the medians of general QoL and domains regarding groups for career end period.

However, in the psychological domain, these differences are more likely to occur (*p* = 0.072). This suggests that factors related to mental health may have an impact on the QoL of Former players, especially in the first few years after their professional career ends. Although there are no significant differences in QoL perception between groups, former players who finished their careers between 2006 and 2018 consistently have lower median values than those who finished between 1998 and 2005 in all domains (Figure 1).

It was not expected that older players would have a higher general QoL, particularly in the physical domain. Former players may face challenges during the transition to post-career [11,58,59], including social adaptation, psychological instability, and changing environmental contexts. Perhaps the better QoL among players who retired longer ago might relate to them having transitioned and adjusted to life after football already, while players who retired more recently might still be adjusting. This idea can be supported by a systematic review that suggests issues of adjustment and mental health during the early years after retirement, while psychological issues apparently declined around 1–2 decades after retirement [19]. Also, approximately 20% of athletes have difficulties at the end of their careers [37]. These difficulties include, among others, feelings of loss, self-confidence problems, identity crisis, anguish, loss of social relationships, the process of adapting to a new social status, financial insecurity, occupational delay and adaptation to a new lifestyle and daily routine [26,60,61,62]. These factors could make the transition to retirement harder for young former players, thus more likely to yield a lower QoL. However, these findings highlight the need to further investigate the link between QoL and health indicators in this population over time.

### 3.3. Quality of Life and Competitive Level

The study sample consisted of professional football players from 1st (51.2%) and 2nd division leagues in Portugal or abroad (48.8%). The results are presented in relation to the QoL scores obtained by both groups (Table 8).

Former players with the highest competitive level, particularly those who dominated the 1st division, have higher mean points in all domains. However, these differences are not statistically significant in the general QoL facet or in the remaining domains (Table 9).

Players’ competitive level during their careers does not appear to affect their perception of QoL thereafter. However, there is a trend in all domains to increase QoL among players who play at a higher competitive level. A similar study found statistically significant differences in physical dominance, particularly among two groups of players from different divisions [63]. This paradigm can be explained by the better working conditions offered to elite players in elite clubs, particularly in terms of the quality of available resources and equipment (materials, space, and logistics), medical assistance, and technical support.

### 3.4. Quality of Life and the Tactical-Position Status 

Based on the tactical positions they had during their careers, the majority of former players were goalkeepers (16.4%), defenders (15.1%), midfielders (15.5%), and forwards (14.8%). Comparisons of general QoL and all domains do not show significant effects based on the tactical-positional status (Table 10).

Consider that QoL in the post-career is not related to the tactical-positional status. Defender players tend to have higher QoL scores (M = 81.4), while goalkeepers have lower QoL scores (M = 73.3) in general. Similarly, in the domains, defenders have higher averages (M = 83.3) compared to goalkeepers (M = 76.4). In fact, various types of sports activity (endurance, resistance, or mixed) may influence QoL in different ways; however, there is a paucity of data regarding the role of different positions played in team sports such as football [64]. In a study that compared the attention performance by playing position in elite Brazilian football players, it was verified that the players of defensive positions, as defenders and midfielders, were more attentive and less impulsive than players of other positions [65]. In another study, goalkeepers were shown to have a more passive role in the game, with fewer technical interventions and less influence on team orientation compared to midfielders and defenders [63]. However, a separate study revealed goalkeepers were characterized by a 5–8-year longer life duration compared to their colleagues playing in other positions, with an absence of differences between defenders, midfielders, and forwards [64]. In this way, it would be consistent for the goalkeepers in our sample to demonstrate a higher level of QoL, which was not the case. So, the trend of the results has to be confirmed with a larger sample size. 

### 3.5. Quality of Life and the International Status

A total of 26.2% of former players represented the Portuguese national “AA” team on an international level. The study aimed to determine whether the QoL perceptions of former international players differed from those of non-international players (73.8%). The comparison did not show statistically significant differences in the general aspect of QoL or in the remaining domains (Table 11).

**Figure 2:**
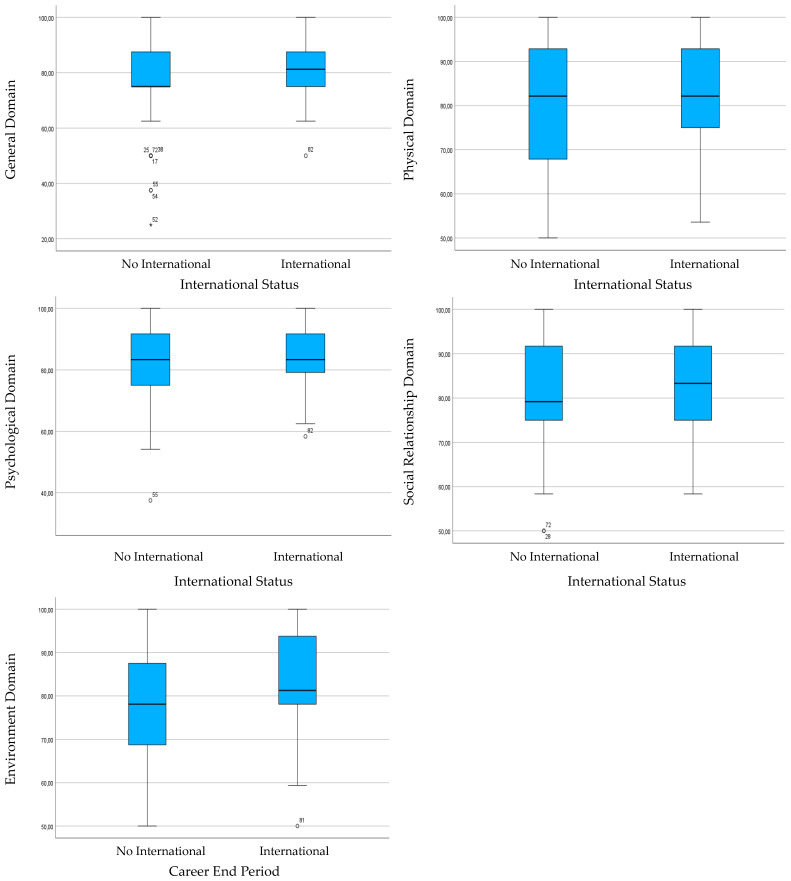
Comparison of the medians of general QoL and domains regarding groups for international status.

However, based on the average values of each domain (Figure 2), it is evident that former international players had a higher QoL than non-international players, with few exceptions.

There are few references to support this trend; however, a retrospective study of the careers of Portuguese players shows that internationals have a more involuntary transition and struggle to adjust to the post-career period, particularly in the first year of reform [58]. Similar results have been seen in athletes from other sports [23,66], indicating that the QoL of these former players may be slightly lower, particularly in the psychological, social, and environmental domains. These previous findings may be related to the strong athletic identity of international-level players [20,67]. However, this notion does not align with the trend observed in our analyses, and it should be noted that past studies did not directly compare international athletes with non-international athletes.

### 3.6. Quality of Life and Academic Qualifications

When the former players were interviewed in post-career, 36% had literacy skills up to the third cycle or lower, 34.5% high school education, and 28.6% had university education. 

First, it is worth noting the high academic qualifications of the inquired former players when compared to other studies, which reported that less than 10% of former players had a university education certificate [28,58,68]. Taking into account the defined levels, QoL was compared based on academic qualifications. In the first analysis (Table 12), it is observed that the scores obtained in the general QoL and in various domains are increasing in proportion to the abilities; that is, former players with higher education levels have better results for the general QoL (82.2 ± 18.02) than those with the third cycle (74.5 ± 15.3).

The results (Table 13) showed that there are statistically significant differences in QoL between the general QoL and the physical domain of QoL for the holders of the various academic qualifications throughout the post-career transition.

A multiple-comparison pairwise test was used to verify at what levels differences occur in general QoL and the physical domain of QoL (Table 14 and Table 15). So, these differences were found between university education and the third cycle education in general QoL and the physical domain of QoL. Simultaneously, there were differences in third-cycle education and high school education in physical domain of QoL. 

Empirically, it is considered that formation is a means of excellence for personal and professional growth in any area of activity [7]. The acquisition of lifelong formation in football can be a determining factor in making players better prepared for the challenges they encounter in post-career life [69]. Based on these findings, it is safe to say that the academic qualifications of former players have an impact on QoL that respects the physical domain, raising the question of why they occur only in this domain and not, for example, in the psychological domain. Since it is difficult to reconcile studies with professional careers [34], it was anticipated that former players who obtained higher qualifications could present more positive feelings of self-realization or self-esteem. Higher formal qualifications, however, might imply that players have more retirement alternatives and a higher quality of life than players with lower qualifications, who could find it harder to join another line of work [51,70,71,72]. The results can support this argument mainly due to the difference in general QoL, and the physical domain of QoL found between former players with third-cycle education and university education.

### 3.7. Quality of Life and Severe Career Injuries

A total of 86.9% of former players experienced at least one severe injury during their career, while 13.1% did not. The most common anatomical injury (53.8% of total injuries) occurred at the knee level (Table 16).

Pearson’s correlation coefficient (R = −0.300; *p* = 0.006) indicates a statistically significant relationship between the physical domain of QoL and the number of severe injuries in a career (Table 17).

Severe injuries during a career are a significant factor in long-term health and in QoL [14,23,48,54], and the findings of our study confirm this trend. In fact, the football career is short, and injuries are one of the common worries players have throughout their careers [27,73]. The sense of identity marked in the “physical” body directs this phenomenon to something equivalent to trauma or chronic illness [74] since professional status depends on the player’s physical-athletic ability [75,76]. Football is a contact sport played at high speed, so it presents a high risk of injury incidence [77], which must be considered a key element for a long-term QoL in the physical domain. 

### 3.8. Quality of Life and Current Professional Connection to Football

A total of 60.7% of former players were professionally involved in football as coaches, directors, or agents. A total of 39.3% had no professional connection to the football. There are no statistically significant differences between these groups, whether in the general aspect of QoL or in the remaining domains (Table 18).

In terms of the environmental domain, the differences are close to statistical significance (*p* = 0.068). According to the literature, this type of employment has an important role in ensuring a better life since it meets human needs, particularly those of self-esteem, financial security, and social security [78,79,80]. Despite the results, it is worth pointing out in other studies the importance of professional work to the identity of the former players when retired [58,69], because the work may influence the notion of self and self-worth. 

## 4. Conclusions

This article uses a broad perspective on QoL in relation to social sciences to contribute to the field of health, specifically the impact of sports careers on the QoL of former football players. The objective was to assess the QoL of former Portuguese football players using the WHOQOL-BREF. A total of 84 former players (48.8 ± 8.2 years) were interviewed.

Based on the results of the general QoL assessment and the relevant domains (physical, psychological, social, and environmental), it was found that there are statistically significant differences in QoL based on the duration of the career, the period of end career, the competitive level, the tactical-positional status, the international status, the academic qualifications, the severe career injuries, and the current professional connection to football. The results show that former players have a good general QoL (78.7 ± 15.7), exceeding initial expectations based on comparable studies conducted with football players [14,26,28,29,58,80]. This trend is observed in all QoL domains, including physical (81.1 ± 13.1), psychological (82.1 ± 12.8), social relationships (81.5 ± 12.6), and environment (79.3 ± 12.3). These indicators support the idea that a professional career can provide long-term benefits in QoL, including not just physical activity but also living conditions, status, social support, and available resources. When comparing the results of QoL general and various domains with the variables analyzed, there are no significant findings in almost any variable. Exceptions have occurred at the level of academic qualifications, in which it is noted that the higher the qualification, the better the general QoL (*p* < 0.001) and the physical domain (*p* = 0.023) and, furthermore, in the occurrence of severe career injuries that were negatively associated with the physical domain of QoL (*p* = 0.006). This is a crucial conclusion to reinforce the need for academic formation throughout life, as well as investing in safety, prevention, and intervention in sports injuries.

To conclude, it was determined that there are no concerning findings at the level of QoL given by former players based on the evidence of the results. It is important to note, however, that taking into account the type of studied population, the conclusions may show limitations not only from the reduced sample (*n* = 84) but also from their convenience selection that included 60.7% of former players being professionally connected to football.

Assessing the QoL of former players is crucial for identifying and characterizing the long-term impact of the career. This allows for the development of preventive and intervention strategies (pre, during, and post-career) to maximize the QoL of this population after leaving the career.

## Figures and Tables

**Table 1:** Current residences of former players included in the sample.

Regions (Nuts II)	N	%
North	15	17.9
Center	25	29.8
Lisbon and Vale do Tejo	27	32.1
Alentejo	6	7.1
Algarve	3	3.6
Madeira e Açores	6	7.1
Out from Portugal	2	2.4
Total	84	100.0

**Table 2:** Structure adapted from WHOQOL-BREF: domains and facets [43].

	Domains	Facets
WHOQOL-BREF		General Quality of Live
	Physical	Pain and Discomfort Energy and Fatigue Sleep and Rest Mobility Activities of Daily Living Dependence on Medication or Treatments Work Capacity
	Psychological	Positive feelings Thoughts, feelings, memory and concentration Self-esteem Body image and appearance Negative feelings Spirituality/Religion/Beliefs
	Social relationships	Personal relationships Social support Sexual activity
	Environment	Physical security Home environment Economic Resources Health and social care Opportunities to acquire new information and skills Participation or opportunities for recreation and leisure Physical environment (pollution/noise/traffic/climate) Transport

**Table 3:** Transformation of the WHOQOL-BREF domain results on a scale of 0 to 100.

WDG = Sum.2 (Q1, Q2)
WD1 = Sum.7 (Q3, Q4, Q10, Q15, Q16, Q17, Q18)
WD2 = Sum.6 (Q5, Q6, Q7, Q11, Q19, Q26)
WD3 = Sum.3 (Q20, Q21, Q22)
WD4 = Sum.8 (Q8, Q9, Q12, Q13, Q14, Q23, Q24, Q25)
MDG = 4 × (Mean(Q1, Q2))
MD1 = 4 × (Mean(Q3, Q4, Q10, Q15, Q16, Q17, Q18))
MD2 = 4 × (Mean(Q5, Q6, Q7, Q11, Q19, Q26))
MD3 = 4 × (Mean(Q8, Q9, Q12, Q13, Q14, Q23, Q24, Q25))
TWDG = ((WDG–2)/8) × 100
TWD1 = ((WD17)/28) × 100
TWD2 = ((WD2–6)/24) × 100
TWD3 = ((WD3–3)/12) × 100
TWD4 = ((WD4–8)/32) × 100

**Table 4:** Minimum values, maximum values, means and standard deviations of QoL scores.

	N	Minimum	Maximum	Mean	Std Deviation
General QoL	84	25.00	100.00	78.7202	15.73737
Physical Domain	84	50.00	100.00	81.1650	13.13516
Psychological Domain	84	37.50	100.00	82.0933	12.85115
Social Relationship Domain	84	50.00	100.00	81.5476	12.67920
Environment Domain	84	50.00	100.00	79.3899	12.37816

**Table 5:** Differences in quality of life based on career duration (Pearson correlation).

		Career Duration	General QoL	Physical Domain	Psychological Domain	Social Relationships Domain	Environmental Domain
Career duration	Pearson correlation	1	−0.105	−0.080	−0.136	−0.076	0.009
	Sig. (2 ext.)		0.342	0.469	0.218	0.491	0.937
	N	84	84	84	84	84	84

**Table 6:** Mean and standard deviation of the general QoL score depending on career end period.

	Career End Period	N	Mean	Std Deviation
General QoL	1988–2005	33	79.1667	13.13492
	2006–2018	51	78.4314	17.33465

**Table 7:** Significance of differences in QoL and career end period (Student’s *t*-test).

	t	df	Sig
General QoL (TWDG)	0.208	82	0.836
Physical Domain (TWD1)	0.486	82	0.628
Psychological Domain (TWD2)	1.823	82	0.072
Social Relationship Domain (TWD3)	1.039	82	0.302
Environment Domain (TWD4)	0.711	82	0.479

**Table 8:** Means and standard deviations of general QoL scores and domains by competitive level.

		N	Mean	Std. Deviation	Std. Error Mean
General QoL	1st division	43	81.1047	12.00573	1.83086
	2nd division	41	76.2195	18.70931	2.92190
Physical Domain	1st division	43	82.3920	13.05022	1.99014
	2nd division	41	79.8780	13.26140	2.07108
Psychological Domain	1st division	43	84.0116	12.98434	1.98009
	2nd division	41	80.0813	12.55240	1.96036
Social Relations Domain	1st division	43	83.5271	12.26477	1.87036
	2nd division	41	79.4715	12.92306	2.01824
Environment Domain	1st division	43	81.7587	13.06268	1.99204
	2nd division	41	76.9055	11.24301	1.75586

**Table 9:** Comparative analysis between QoL and competitive level (Student’s *t*-test).

	t	df	Sig
General QoL	1.431	82	0.156
Physical Domain	0.876	82	0.384
Psychological Domain	1.409	82	0.162
Social Relationship Domain	1.476	82	0.144
Environment Domain	1.821	82	0.072

**Table 10:** Significance differences of QoL and tactical-positional status (One-way ANOVA).

		Sum of Squares	df	Mean Square	F	Sig.
General QoL	Between Groups	943.411	3	314.470	1.283	0.286
	Within Groups	19,612.765	80	245.160		
	Total	20,556.176	83			
Physical Domain	Between Groups	690.446	3	230.149	1.351	0.264
	Within Groups	13,629.738	80	170.372		
	Total	14,320.183	83			
Psychological Domain	Between Groups	245.660	3	81.887	0.487	0.693
	Within Groups	13,461.971	80	168.275		
	Total	13,707.631	83			
Social Relationships Domain	Between Groups	789.609	3	263.203	1.677	0.178
	Within Groups	12,553.645	80	156.921		
	Total	13,343.254	83			
Environment Domain	Between Groups	284.464	3	94.821	0.610	0.610
	Within Groups	12,432.705	80	155.409		
	Total	12,717.169	83			

**Table 11:** Comparative analysis between QoL and international status (Student’s *t*-test).

	t	df	Sig
General QoL	−1.277	82	0.205
Physical Domain	−0.472	82	0.638
Psychological Domain	−0.928	82	0.356
Social Relationship Domain	−1.263	82	0.210
Environment Domain	−1.392	82	0.168

**Table 12:** Medians and interquartile range of QoL in terms of academic qualifications.

	Academic Qualification					
	Third Cycle or Lower		High School		University Education	
	Median	IQR	Median	IQR	Median	IQR
General QoL	75.0000	12.50	75.0000	12.50	87.5000	25.00
Physical Domain	75.0000	14.29	85.7143	26.79	92.8571	17.86
Psychological Domain	83.3333	12.50	87.5000	22.92	83.3333	12.50
Social Relationship Domain	75.0000	16.67	83.3333	20.83	83.3333	22.92
Environment Domain	78.1250	18.75	81.2500	23.44	79.6875	15.63

**Table 13:** Significance of QoL differences in academic qualifications (Jonckheere–Terpstra Test).

	General QoL	Physical Domain	Psychological Domain	Social Relationships Domain	Environmental Domain
N Academic Qual. Levels	3	3	3	3	3
N	84	84	84	84	84
J-T Observed Statistics	1432.000	1588.500	1288.000	1357.500	1237.000
J-T Statistics Average	1169.500	1169.500	1169.500	1169.500	1169.500
J-T Statistics Dev test stat	115.360	121.035	120.810	118.551	120.992
Test Statistics J-T Stat	2.275	3.462	0.981	1.586	0.558
Significance Sig. (2 ext.)	0.023	<0.001	0.327	0.113	0.577

**Table 14:** Multiple comparison pairwise tests between general QoL and academic habilitations.

Sample 1-Sample 2	Test Statistics	Standard Error	Standard Test Statistics	Sig.	Adj. Sig.
Third cycle or lower—High school	538.000	63.205	1.400	0.081	0.242
Third cycle or lower—University education	492.000	56.400	2.128	0.017	0.050
High school—University education	402.000	53.324	1.013	0.156	0.467

**Table 15:** Multiple comparison pairwise tests between physical domain QoL and academic habilitations.

Sample 1-Sample 2	Test Statistics	Standard Error	Standard Test Statistics	Sig.	Adj. Sig.
Third cycle or lower—High school	595.500	67.317	2.169	0.015	0.045
Third cycle or lower—University education	569.500	58.615	3.369	<0.001	0.001
High school—University education	423.500	55.660	1.356	0.087	0.262

**Table 16:** Type, number and percentage of severe career injuries of former players.

		N	%
Injuries	Ankle	13	8.1%
	Leg	7	4.4%
	Knee	86	53.8%
	Thigh	9	5.6%
	Hip/groin	10	6.3%
	Back	5	3.1%
	Head/face/nose	7	4.4%
	Others	23	14.4%
Total		160	100.0%

**Table 17:** Significance of differences between QoL and severe career injuries (Pearson correlation).

		General QoL	Physical Domain	Psychological Domain	Social Relationships Domain	Environmental Domain	Serious Injuries
	N	84	84	84	84	84	84
Serious Injuries	Pearson Corr	−0.109	−0.300 **	−0.148	−0.177	−0.115	1
	Sig. (two-tailed)	0.323	0.006	0.178	0.107	0.296	
	N	84	84	84	84	84	84

** Correlation is significant at the 0.01 level (two-tailed).

**Table 18:** The significance of differences between QoL and current professional connection to football (Student’s *t*-test).

	t	df	Sig
General QoL	−1.033	82	0.304
Physical Domain	−1.466	82	0.147
Psychological Domain	−0.881	82	0.381
Social Relationship Domain	−0.869	82	0.387
Environment Domain	−1.851	82	0.068

## Data Availability

The study database can be provided by the corresponding author.

## References

[1] Amorim M., Coelho R. (1999). Saúde, doença e qualidade de vida. Psiquiatr. Clínica.

[2] Cheik N.C., Reis I.T., Heredia R.A.G., de Ventura M.L., Tufik S., Antunes H.K.M., De Mello M.T. (2003). Efeitos do exercício físico e da atividade física na depressão e ansiedade em indivíduos idosos. Rev. Bras. Ciência Mov..

[3] Gutierrez G.L. (2012). Qualidade de Vida: Definição, Conceito e Interfaces Com Outras Áreas de Pesquisa.

[4] Pereira É.F., Teixeira C.S., dos Santos A. (2012). Qualidade de vida: Abordagens, conceitos e avaliação. Rev. Bras. Educ. Física Esporte.

[5] de Minayo M.C.S., de Hartz Z.M.A., Buss P.M. (2000). Qualidade de vida e saúde: Um debate necessário. Ciência Saúde Coletiva.

[6] Rodrigues J., Chicau Borrego C., Ruivo P., Sobreiro P., Catela D., Amendoeira J., Matos R. (2020). Conceptual Framework for the Research on Quality of Life. Sustainability.

[7] Patrício B., Jesus L.M.T., Cruice M., Hall A. (2014). Quality of Life Predictors and Normative Data. Soc. Indic. Res..

[8] Pais-Ribeiro J.L. (2004). Quality of life is a primary end-point in clinical settings. Clin. Nutr..

[9] Seidl E.M.F., da C Zannon C.M.L. (2004). Qualidade de vida e saúde: Aspectos conceituais e metodológicos. Cad. Saúde Pública.

[10] Renwick R., Brown I., Nagler M. (1996). Quality of Life in Health Promotion and Rehabilitation: Conceptual Approaches, Issues, and Applications.

[11] Samuel R.D., Stambulova N., Galily Y., Tenenbaum G. (2023). Adaptation to change: A meta-model of adaptation in sport. Int. J. Sport. Psychol..

[12] Haraldstad K., Wahl A., Andenæs R., Andersen J., Andersen M.H., Beisland E., Borge C., Engebretsen E., Eisemann M., Halvorsrud L. (2019). A systematic review of quality of life research in medicine and health sciences. Qual. Life Res..

[13] WHOQOL Group (1995). The World Health Organization quality of life assessment (WHOQOL): Position paper from the World Health Organization. Soc. Sci. Med..

[14] Arliani G.G., Astur D.C., Yamada R., Yamada A.F., da Rocha Corrêa Fernandes A., Ejnisman B., Castro Pochini A., Cohen M. (2016). Professional football can be considered a healthy sport?. Knee Surg. Sports Traumatol. Arthrosc..

[15] Barth M., Emrich E. (2021). Retirement of professional soccer players—A systematic review from social sciences perspectives. J. Sports Sci..

[16] Filbay S., Pandya T., Thomas B., McKay C., Adams J., Arden N. (2019). Quality of Life and Life Satisfaction in Former Athletes: A Systematic Review and Meta-Analysis. Sports Med..

[17] Maffulli N., Longo U.G., Gougoulias N., Caine D., Denaro V. (2011). Sport injuries: A review of outcomes. Br. Med. Bull..

[18] Stambulova N., Alfermann D., Statler T., Côté J. (2009). ISSP Position stand: Career development and transitions of athletes. Int. J. Sport. Exerc. Psychol..

[19] Stambulova N.B., Ryba T.V., Henriksen K. (2021). Career development and transitions of athletes: The International Society of Sport Psychology Position Stand Revisited. Int. J. Sport. Exerc. Psychol..

[20] Monteiro R., Monteiro D., Torregrossa M., Travassos B. (2021). Career Planning in Elite Soccer: The Mediating Role of Self-Efficacy, Career Goals, and Athletic Identity. Front. Psychol..

[21] Barreira D., Garganta J., Castellano J., Machado J., Anguera M.T. (2015). How elite-level soccer dynamics has evolved over the last three decades?: Input from generalizability theory. Cuad. Psicol. Deporte.

[22] Carapinheira A., Mendes P., Guedes Carvalho P., Travassos B. (2019). Sports Career termination in football players: Systematic review. Rev. Iberoam. Psicol. Ejerc. Deporte.

[23] Knights S., Sherry E., Ruddock-Hudson M., O’Halloran P. (2019). The End of a Professional Sport Career: Ensuring a Positive Transition. J. Sport Manag..

[24] Lelbach A., Dörnyei G., Ihász F., Koller A. (2020). Post-sports career healthy ageing: The Janus-faced, high-performance sport. Dev. Health Sciences.

[25] Carmody S., Anemaat K., Massey A., Kerkhoffs G., Gouttebarge V. (2022). Health conditions among retired professional footballers: A scoping review. BMJ Open Sport Exerc. Med..

[26] Carmody S., Aoki H., Kilic O., Maas M., Massey A., Kerkhoffs G., Gouttebarge V. (2022). Osteoarthritic changes in the knees of recently retired male professional footballers: A pilot study. S. Afr. J. Sports Med..

[27] Zech A., Wellmann K. (2017). Perceptions of football players regarding injury risk factors and prevention strategies. PLoS ONE.

[28] Carapinheira A., Mendes P., Guedes Carvalho P., Torregrossa M., Travassos B. (2018). Career Termination of Portuguese Elite Football Players: Comparison between the Last Three Decades. Sports.

[29] Drawer S., Fuller C.W. (2002). Perceptions of retired professional soccer players about the provision of support services before and after retirement. Br. J. Sports Med..

[30] Drawer S., Fuller C.W. (2001). Propensity for osteoarthritis and lower limb joint pain in retired professional soccer players. Br. J. Sports Med..

[31] Stephan Y., Bilard J., Ninot G., Delignieres D. (2003). Repercussions of Transition Out of Elite Sport on Subjective Well-Being: A One-Year Study. J. Appl. Sport Psychol..

[32] Maseko J., Surujlal J. (2011). Retirement planning among South African professional soccer players: A qualitative study of players’ perceptions: Job satisfaction sport. Afr. J. Phys. Health Educ. Recreat. Danc..

[33] Selingardi D.C. (2013). Término e Recomeço: Da Carreira atlética à Aposentadoria. Master’s Thesis.

[34] Wylleman P., Alfermann D., Lavallee D. (2004). Career transitions in sport: European perspectives. Psychol. Sport Exerc..

[35] Martin L.A., Fogarty G.J., Albion M.J. (2014). Changes in Athletic Identity and Life Satisfaction of Elite Athletes as a Function of Retirement Status. J. Appl. Sport Psychol..

[36] Brandão M.R., Akel M.C., Andrade A., Guiselini M., Martini L., Nastás M. (2000). Causas e conseqüências da transição de carreira esportiva: Uma revisão de literatura. Rev. Bras. Ciência Mov..

[37] Park S., Lavallee D., Tod D. (2013). Athletes’ career transition out of sport: A systematic review. Int. Rev. Sport Exerc. Psychol..

[38] Berger B.G., Eklund R.C., Weinberg R.S. (2015). Foundations of Exercise Psychology.

[39] Raphael D., Brown I., Renwick R., Rootman I. (1996). Assessing the quality of life of persons with developmental disabilities: Description of a new model, measuring instruments, and initial findings. Int. J. Disabil. Dev. Educ..

[40] WHOQOL Group (1994). Development of the WHOQOL: Rationale and Current Status. Int. J. Ment. Health.

[41] World Health Organization (1997). Division of Mental Health and Prevention of Substance Abuse WHOQOL: Measuring Quality of Life.

[42] Vaz Serra A., Canavarro M.C., Simões M., Pereira M., Gameiro S., Quartilho M.J., Rijo D., Carona C., Paredes T. (2006). Estudos psicométricos do instrumento de avaliação da qualidade de vida da Organização Mundial de Saúde (WHOQOL-Bref) para Português de Portugal. Psiquiatr. Clínica.

[43] Canavarro M., Serra A., Pereira M., Simões M., Quintais l., Quartilho M.J., Rijo D., Carona C., Gameiro S., Paredes T. (2006). Desenvolvimento do Instrumento de Avaliação e Qualidade de Vida da Organização Mundial da Saúde (WHOQOL-100) para Português de Portugal. Psiquiatr. Clínica.

[44] Canavarro M.C., Moreira H., Paredes T. (2010). Qualidade de vida e saúde: Aplicações do WHOQOL. Alicerces.

[45] Kluthcovsky A.C.G.C., Kluthcovsky F.A. (2009). O WHOQOL-bref, um instrumento para avaliar qualidade de vida: Uma revisão sistemática. Rev. Psiquiatr. Rio Gd. Sul..

[46] Teixeira E., Silva C., Vicente A. (2024). Development and validation of an interview guide for examining the effects of sports careers on the quality of life of retired Portuguese football players. Front. Psychol..

[47] Soares J. (2007). O Treino do Futebolista—Volume 2. Lesões. Nutrição.

[48] Ekstrand J., Hägglund M., Waldén M. (2011). Epidemiology of Muscle Injuries in Professional Football (Soccer). Am. J. Sports Med..

[49] Ekstrand J., Karlsson J. (2003). The risk for injury in football. There is a need for consensus about definition of the injury and the design of studies. Scand. J. Med. Sci. Sports.

[50] Ho R. (2014). Handbook of Univariate and Multivariate Data Analysis with IBM SPSS.

[51] Barth M., Schlesinger T., Pitsch W. (2022). Is Professional Soccer a Risk for Their “Lives Afterwards”? A Social-Sciences-Based Examination of Retired Professional Soccer Players from a Long-Term Perspective. J. Risk Financ. Manag..

[52] Fernandes G.S., Parekh S.M., Moses J., Fuller C.W., Scammell B., Batt M.E., Zhang W., Doherty M. (2019). Depressive symptoms and the general health of retired professional footballers compared with the general population in the UK: A case–control study. BMJ Open.

[53] Gouttebarge V., Frings-Dresen M.H.W., Sluiter J.K. (2015). Mental and psychosocial health among current and former professional footballers. Occup. Med..

[54] Gouttebarge V., Aoki H., Ekstrand J., Verhagen E.A., Kerkhoffs G.M. (2016). Are severe musculoskeletal injuries associated with symptoms of common mental disorders among male European professional footballers?. Knee Surg. Sports Traumatol. Arthrosc. Off. J. ESSKA.

[55] Jones A., Jones G., Greig N., Bower P., Brown J., Hind K., Francis P. (2021). Corrigendum to’ Epidemiology of injury in English professional football players: A cohort study’ [Physical Therapy in Sport 35C (2018)18–22]. Phys. Ther. Sport.

[56] Fleck M. (2008). Problemas conceituais em qualidade de vida. Avaliação da Qualidade de Vida: Guia Para Profissionais de Saúde.

[57] Nosraty L., Deeg D., Raitanen J., Jylhä M. (2023). Who live longer than their age peers: Individual predictors of longevity among older individuals. Aging Clin. Exp. Res..

[58] Carapinheira A., Torregrossa M., Mendes P., Guedes Carvalho P., Travassos B.F.R. (2018). A retrospective analysis of retirement of football players in Portugal. Motricidade.

[59] Taylor J., Ogilvie B.C. (1994). A conceptual model of adaptation to retirement among athletes. J. Appl. Sport Psychol..

[60] Gouttebarge V., Aoki H., Kerkhoffs G. (2018). Lower extremity osteoarthritis is associated with lower health-related quality of life among retired professional footballers. Phys. Sportsmed..

[61] van Ramele S., Aoki H., Kerkhoffs G.M.M.J., Gouttebarge V. (2017). Mental health in retired professional football players: 12-month incidence, adverse life events and support. Psychol. Sport Exerc..

[62] Van Yperen N. (2009). Why Some Make It and Others Do Not: Identifying Psychological Factors That Predict Career Success in Professional Adult Soccer. Sport Psychol..

[63] Moisao A., Brito-Costa C., Vicente Castro C., Amorim S.A.H., Fernandéz M.I. (2016). Qualidade de vida em atletas de futebol. Rev. INFAD Psicología. Int. J. Dev. Educ. Psychol..

[64] Śmigielski W., Gajda R., Małek Ł., Drygas W. (2020). Goalkeepers Live Longer than Field Players: A Retrospective Cohort Analysis Based on World-Class Football Players. Int. J. Environ. Res. Public Health.

[65] Moreira L., Malloy-Diniz L.F., Pinheiro G.S., Costa V.T. (2022). Are there differences in the attention of elite football players concerning playing positions?. Sci. Med. Footb..

[66] Hatamleh M.R. (2013). The life transitions of hig performance athletes retirement from sport. Eur. Sci. J..

[67] McKnight K., Bernes K., Gunn T., Chorney D., Orr D., Bardick A. (2009). Life After Sport. Atletic Career Transition and Transferable Skills. J. Excell..

[68] Curran C. (2015). Post-playing careers of Irish-born footballers in England, 1945–2010. Sport Soc..

[69] Breslin D.G., Ferguson M.K., Shannon D.S., Haughey M.T., Connor S. (2019). Player Transition Out of Football to Protect Wellbeing: A Dual Career Identity Study.

[70] Ehnold P., Gohritz A., Lotzen L., Schlesinger T. (2024). Soccer above all? Analysis of academic and vocational education among female soccer players in the German women’s Bundesliga and 2nd women’s Bundesliga. Front. Sports Act. Living.

[71] Vilanova A., Puig N. (2017). Estrategias de entrada al mercado de trabajo de los atletas olímpicos. Una tipología. Rev. Int. Sociol..

[72] Smismans S., Wylleman P., De Brandt K., Defruyt D. (2021). From elite sport to the job market: Development and initial validation of the Athlete Competency Questionnaire for Employability (ACQE) (Del deporte de élite al mercado laboral: Desarrollo y validación inicial del Cuestionario de Competencias de Deportistas para la Empleabilidad (ACQE)). Cult. Cienc. Deporte CCD.

[73] Turner D. (2010). Qualitative Interview Design: A Practical Guide for Novice Investigators. Qual. Rep..

[74] Charmaz K., Albrecht G.L., Fitzpatrick R., Scrimshaw S.C. (2003). Experiencing chronic illness. Handbook of Social Studies in Health and Medicine.

[75] Melekoğlu T., Sezgin E., Işın A., Türk A. (2019). The Effects of a Physically Active Lifestyle on the Health of Former Professional Football Players. Sports.

[76] Roderick M. (2012). An Unpaid Labor of Love: Professional Footballers, Family Life, and the Problem of Job Relocation. J. Sport Soc. Issues.

[77] Pfirrmann D., Herbst M., Ingelfinger P., Simon P., Tug S. (2016). Analysis of Injury Incidences in Male Professional Adult and Elite Youth Soccer Players: A Systematic Review. J. Athl. Train..

[78] Benson S.G., Dundis S.P. (2003). Understanding and motivating health care employees: Integrating Maslow’s hierarchy of needs, training and technology. J. Nurs. Manag..

[79] Luo J., Liu X.-B., Yao Q., Qu Y., Yang J., Lin K., Pan S.R., Wang T., Dai Y., Chen H. (2024). The relationship between social support and professional identity of health professional students from a two-way social support theory perspective: Chain mediating effects of achievement motivation and meaning in life. BMC Med. Educ..

[80] Arliani G.G., Lara P.S., Astur D.C., Cohen M., Gonçalves J.P.P., Ferretti M. (2014). Impact of sports on health of former professional soccer players in Brazil. Acta Ortop. Bras..

